# Computational Protein Redesign of Bacteriophages by Using Evolutionary Algorithms

**DOI:** 10.3390/biology15130997

**Published:** 2026-06-25

**Authors:** Rolando Armas, Ariel Pincay, Cristofer Motoche-Monar, Francisco Hidrobo, José A. Castillo

**Affiliations:** 1 School of Mathematical and Computational Sciences, Yachay Tech University, Urcuqui 100115, Ecuador; tarmas@yachaytech.edu.ec (R.A.); ariel.pincay@yachaytech.edu.ec (A.P.); fhidrobo@yachaytech.edu.ec (F.H.); 2 Phage Therapy Group, Yachay Tech University, Urcuqui 100115, Ecuador; cristofer.motoche@yachaytech.edu.ec; 3 School of Biological Sciences and Engineering, Yachay Tech University, Urcuqui 100115, Ecuador

**Keywords:** protein–protein interaction, protein redesign, evolutionary algorithms, physic-based models, data-driven models

## Abstract

Protein–protein interactions are a fundamental component of most biological processes in living organisms. Therefore, enhancing these molecular interactions is of major importance in the life sciences field. In this paper we present the redesign of a protein–protein complex using single-elitist and multi-objective evolutionary algorithms. We focus on a structural complex composed of a bacteriophage protein interacting with a bacterial protein, emphasizing the interaction zone, which is defined by the closest distance between residues from proteins and comprises thirty-eight positions. Our approach fuses physics-based energy calculations with data-driven models to maximize the effectiveness of the search process. By simultaneously minimizing interaction energy and maximizing the log-likelihood ratio, the proposed algorithms show a balance between thermodynamic stability and biological sequence plausibility. This hybrid strategy guides the mutation of specific residues, enabling the identification of optimal solutions that are both physically robust and evolutionarily relevant. Results demonstrate that the evolved Pareto optimal set exhibits a significant improvement in binding affinity, with mean interface energy decreasing from +32 to −60 REU (Rosetta Energy Units). Furthermore, the analysis identifies key conserved residue positions, validating the capability of the framework to produce energetically favorable and biologically consistent protein designs.

## Simple Summary

Proteins in living organisms often act by binding to other proteins, and scientists seek to improve these interactions to better understand biological processes and develop new biotechnological applications. In this study, we redesigned the interaction between two proteins: one from a bacteriophage and the other from a plant pathogenic bacterial species, with the ultimate goal of achieving more efficient control of this pathogen. We focused on the interaction regions between the two proteins. To improve this interaction, we used evolutionary algorithms that combine physics-based energy calculations with data-driven models. The algorithms were guided to find solutions that were both energetically stable and biologically realistic. As a result, we obtained redesigned protein variants with a much stronger binding capacity between the two proteins. The interactive energy improved substantially, indicating a more stable complex. We also identified key positions at the interface that remain important for maintaining function. Overall, our method provides an efficient computational strategy for designing protein–protein interactions that are both physically robust and biologically meaningful.

## 1. Introduction

Over millions of years, natural evolution has proven effective in creating a wide range of life forms. It plays a crucial role in the development and adaptation of proteins, which are essential building blocks of life. Organisms have evolved diverse protein structures and functions that enable them to succeed in their specific environments. These proteins, formed by the precise arrangement of amino acids, have adapted to facilitate critical processes such as metabolism, immune response, and cell signaling. Through mechanisms such as mutation, natural selection, and genetic drift, certain protein variations have proven advantageous, thereby enhancing an organism’s survival and reproductive success [1]. Inspired by these natural processes, researchers have sought computational approaches that can emulate such evolutionary mechanisms. Evolutionary algorithms (EAs) simulate natural processes in computational environments by using a population of candidate solutions that mimic evolution over time [2]. Just as natural evolution shapes protein structures and functions through selection and variation, EAs can be applied to model or redesign proteins by emulating these evolutionary principles computationally.

Redesigning proteins with specific functions presents a significant challenge due to the vast combinatorial space of possible amino acid sequences and structures. This search space is so large that it is computationally infeasible to evaluate every possible protein variant. As a result, finding optimal or even viable protein redesigns requires efficient strategies to navigate this complexity. EAs offer a promising approach to this problem by mimicking natural selection, enabling the exploration of large search spaces and the identification of high-performing protein candidates [3].

To address the complexities of protein engineering, methods based on data-driven and physics-based approaches have become essential tools. Data-driven approaches, such as machine learning models, leverage the growing availability of protein sequence and structure databases. By learning from these large datasets, these methods can rapidly predict protein properties and guide the redesign process, particularly when ample high-quality data is available [4,5,6]. Their speed and scalability make them well-suited for navigating vast search spaces, although their accuracy can be limited by dataset biases and gaps. On the other hand, physics-based methods rely on fundamental principles of chemistry and physics, using energy calculations and molecular simulations to estimate protein stability and behavior. While these methods provide valuable mechanistic insights, they tend to be computationally demanding. Recognizing the trade-off between the speed of data-driven models and the exactitude of physics-based simulations provides a strong rationale for their hybridization in protein engineering [7,8,9].

In this work, we present a novel framework for optimizing bacteriophage protein–protein binding affinity by fusing evolutionary algorithms, data-driven, and physics-based strategies. In our approach, EAs serve as efficient exploration methods, navigating the vast sequence space to generate diverse protein candidates. We fuse data-driven models, which act as rapid fitness functions and validation tools, with physics-based methods that provide rigorous atomic-level energy calculations. This fusion ensures that the selected designs are not only biologically plausible according to large-scale data but also physically robust. By clearly delineating the roles of each component, our framework enables both broad exploration and rigorous selection, representing a significant advance in computational protein redesign. We applied the aforementioned strategy to increase the affinity of a key protein involved in host bacteriophage recognition, seeking to overcome natural limitations in bacteria-phage interactions like deficient phage adsorption, low phage stability, and reduced bactericidal activity. The modifications introduced into the phage protein will improve phage adsorption to bacteria, thereby boosting phage stability and enhancing bactericidal activity. In this way, we aim to achieve the ultimate goal of controlling pathogenic bacteria.

## 2. Materials and Methods

### 2.1. Overview

To address the protein redesign problem, we implement single- and multi-objective evolutionary algorithms (EAs) with data-driven and physics-based strategies, utilizing the EvolutionaryScale AI Models (ESM) [10,11] and the Rosetta suite [12,13], respectively. In our framework, EAs drive the exploration mechanism, iteratively searching the vast sequence space to generate diverse protein candidates. We combine data-driven models, such as ESM and multiple sequence alignment (MSA) tools, with physics-based methods implemented through Rosetta to create a robust evaluation and guidance system. Data-driven models act as rapid predictors of binding affinity and guides for mutation plausibility, while physics-based methods provide rigorous assessments of energetic stability.

The redesign process begins by defining the search space using the Rosetta interface_energy application [14]. This tool identifies interface regions and calculates pairwise interaction energies to generate lists of amino acids with stabilizing or destabilizing potential. The EA utilizes these physics-derived lists to direct its mutation operator, focusing the search on promising residues. Crucially, the specific replacement amino acid is selected probabilistically based on data-driven insights (MSA or ESM), effectively combining physical structural needs with evolutionary sequence history.

Once a candidate is mutated, we refine the protein structure using a constrained version of the Rosetta FastRelax mover [15] to adjust backbone and sidechain torsion angles. At each iteration, the new candidate complexes undergo a dual evaluation. The Rosetta InterfaceAnalyzer [16] calculates the total interface energy, providing a physics-based fitness value essential for determining thermodynamic stability. Simultaneously, the ESM model computes the ΔLog-likelihood metric, offering a data-driven assessment of sequence plausibility.

**Figure 1:**
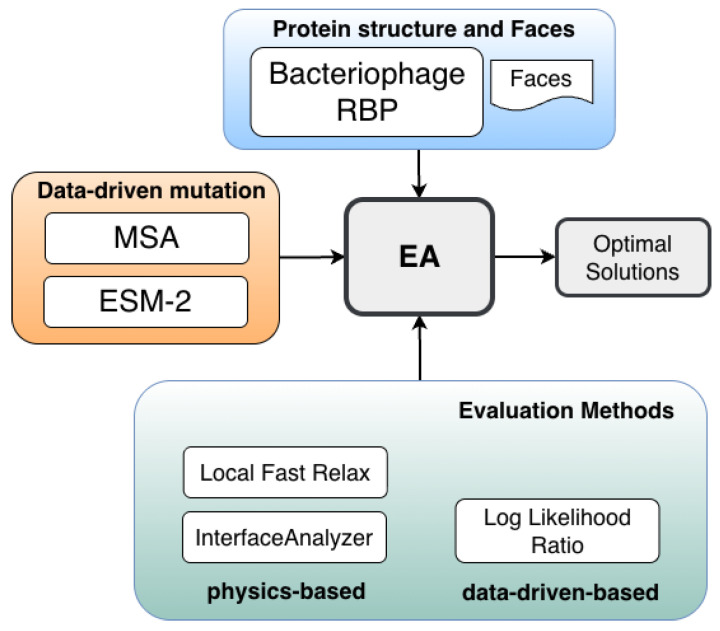
Method and Components: The evolutionary algorithm (EA) takes as inputs the protein structure and its respective faces. The mutation operator is guided by MSA and ESM-2. The fitness functions utilize physics-based (Local Fast Relax, Interface Analyser) and data-driven methods (Log Likelihood Ratio). After running a certain number of generations or iterations, the EA obtains optimal solutions.

This fusion allows for two distinct optimization strategies. The single-objective EA focuses on minimizing the interaction energy to find the most energetically favorable variants. In contrast, the multi-objective EA simultaneously minimizes interaction energy and maximizes the log-likelihood ratio. This approach enables the identification of designs that strike an optimal balance between physical realism and biological plausibility. Figure 1 illustrates the method’s components and their interaction within this framework.

### 2.2. Bacteriophage Proteins as Design Targets

**Figure 2:**
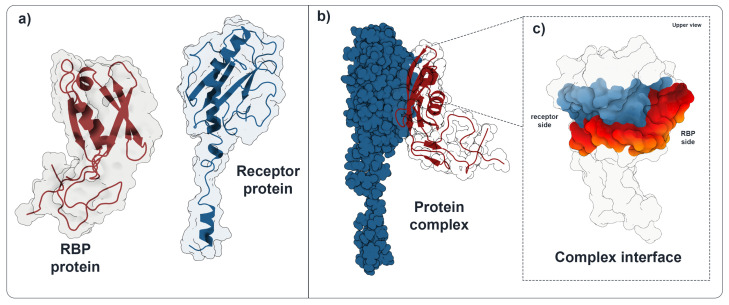
(**a**) AlphaFold2 models obtained after using the default configuration in ColabFold [22]. (**b**) Best-scored complex after one round of molecular docking in GRAMM. We employed the Free Docking methodology, simulated $10^{5}$ scan matches, and used interface residue constraints. The RBP protein was inserted as the ligand. (**c**) A detailed view of the interface created after the docking procedure.

We have performed all the computational analyses using as a model the interaction between phage *ϕ*Rs551 and its host bacterium, Ralstonia solanacearum, the causative agent of bacterial wilt disease of crops [17]. We selected phage *ϕ*Rs551 because it has dual activity in R. solanacearum and can consequently modulate bacterial virulence. In one phase, *ϕ*Rs551 can reduce bacterial infection of plants by repressing host genes involved in virulence [18]. *ϕ*Rs551 recognizes its targets through protein–protein interactions mediated by their Receptor Binding Proteins (RBPs). As a design target, we utilize a protein complex in which the RBP of the bacteriophage Rs551 [17,19] interacts with its respective receptor protein, which is a protein located in the membrane of bacteria. To obtain a completely new and raw complex, we computationally model the separated RBP and receptor proteins using AlphaFold2, a state-of-the-art protein modeling tool [20], and perform a simple round of molecular docking with the GRAMM web server [21], from which we obtain the bound complex. Figure 2 shows a representation of this complex, which comprises two chains A and C, for the receptor (blue) and the RBP (red), respectively.

We will use the Protein Interaction Analysis (PIA) tool incorporated in the Schrödinger Maestro modeling environment [23] to recognize the interacting amino acids in the interface. In this manner, we identified 38 interacting amino acids from the chain C, using a 4 Å distance restraint, and built the Face C file, which contains the positions and identifiers of these amino acids. Face A file contains the information from all the amino acids present in chain A.

### 2.3. MSA Matrix Construction

Amino acid mutations are chosen based on an MSA probability matrix. MSA is the result of sequence alignments of three or more biological sequences, generally DNA or amino acid sequences. These alignments are used to infer evolutionary relationships via phylogenetic analysis and can highlight homologous features between sequences [24]. Therefore, we considered that allowing the evolutionary algorithm to develop in a way that mirrors what has occurred in natural evolution would provide an excellent starting point for our research. Here, we built the MSA of the RBP of the Rs551 bacteriophage. Twenty protein sequences homologous to the Rs551 RBP (with 75–100% identity) were downloaded from the database and aligned with MAFFT [25]. Then, an MSA matrix was created that represents the probability of substitution of each amino acid (row) to another (column). The substitution pattern and rates were estimated under the Jones-Taylor-Thornton model [26]. A discrete Gamma distribution (4 categories) was used to model evolutionary rate differences among sites.

### 2.4. EvolutionaryScale Model (ESM-2)

The ESM-2 transformer model [10] represents a state-of-the-art protein language model that captures deep evolutionary patterns and structural constraints directly from sequence data. Unlike purely physics-based approaches, ESM-2 leverages the statistical properties of millions of observed protein sequences to learn the rules of protein folding and function. This capability allows it to predict the likelihood of specific amino acid occurrences within a sequence context, effectively distinguishing between natural, functional variants and unlikely or deleterious mutations [27].

In our framework, we utilize ESM-2 to infuse biological plausibility into the protein redesign process in two distinct ways. First, it acts as a guide for the mutation operator through the computation of the Log Likelihood Ratio (LLR) Matrix. This matrix quantifies the relative plausibility of substituting a residue at a specific position, allowing the algorithm to prioritize mutations that are evolutionarily viable and structurally supported. Second, ESM-2 serves as a data-driven objective function. By calculating the ΔLog-likelihood metric, we obtain a rapid assessment of sequence plausibility. This dual application effectively combines deep learning-derived evolutionary insights with the search strategy, enhancing the efficiency of exploring the redesign space.

### 2.5. Evolutionary Algorithm

Proteins can fold into countless conformational states. As the sequence length increases, the number of potential structures grows exponentially. Even when limiting the redesign space to the protein–protein interface, exploring the entire combinatorial mutational space remains a formidable challenge. This complexity makes it impractical to exhaustively evaluate all possible sequence variants using traditional methods. Evolutionary algorithms (EAs) are particularly effective in these high-dimensional landscapes, as they efficiently navigate the search space through mechanisms inspired by natural selection. Therefore, we implemented two strategies—single-objective and multi-objective evolutionary algorithms—to simulate the process of molecular evolution and affinity maturation in silico.

The single-objective algorithm employs an elitist strategy to preserve the fittest population member across generations, ensuring that the best energetic variant is never lost. The multi-objective algorithm facilitates the simultaneous optimization of conflicting or complementary goals. In this framework, we optimize both physics-based (stability) and data-driven (plausibility) objective functions. The NSGA-II algorithm [28] is employed to generate a diverse population of candidate solutions, utilizing a non-dominated sorting mechanism and a crowding distance operator to maintain diversity along the Pareto front.

In the following subsections, we describe the solution representation, the mutation operator, and the fitness functions used in this study.

#### 2.5.1. Solution Representation

Let us define *L* as the set of mutable residue positions identified within the Receptor Binding Protein (RBP) interface, denoted as $L=\{l_{1},\;l_{2},\;\ldots ,\;l_{j},\;\ldots ,\;l_{N}\}$. A candidate solution is represented by a linear array of single-letter amino acid codes: $AA_{l_{1}},\;AA_{l_{2}}\cdots ,\;AA_{l_{j}},\;\cdots AA_{l_{N}}$. Each value $AA_{l_{j}}$ represents the specific amino acid residue assigned to position $l_{j}$ in the sequence.

#### 2.5.2. Initial Population

The “Face” file defines the specific subset of amino acid positions (*L*) that comprise the interaction interface. To construct the initial population, we utilize the wild-type sequence at these positions as the template. We then apply the mutation operator to this template to generate a diverse set of initial variants, which constitutes the starting population for the evolutionary process.

#### 2.5.3. Mutation Operator

We implement a specialized mutation operator designed to intelligently traverse the energy landscape. The procedure, detailed in Algorithm 1, modifies the selected gene(s) as follows:

1. Input Processing: The algorithm receives the current population (Pop_g), the list of interface positions (*L*), the mutation rate (*mut_rate*), and the probabilistic guidance matrices: the MSA probability matrix (*MSA_matrix*) and the Log Likelihood Ratio matrix (*LLR_Matrix*).
2. Identification of Target Residues: For a given individual, we first characterize the energetic contribution of each interface residue. Using the Rosetta interface_energy program [14], we classify residues into stabilizing (favorable energy) and destabilizing (unfavorable/high energy) lists (line 2).
3. Defining Mutation Scope: The strategy prioritizes the repair of energetic defects. If the list of destabilizing residues is not empty, it is selected as the target pool; otherwise, the algorithm explores modifications to already stabilizing residues to further enhance affinity (lines 3–7). The maximum number of allowed mutations is derived from the size of the selected pool scaled by the mutation rate (line 8). The actual number of mutations for the current step is chosen stochastically between 1 and this maximum (line 9).
4. Position Selection: Based on the determined number of mutations, specific positions and their current amino acids are selected from the target pool (destabilizing or stabilizing) for substitution (line 11).
5. Amino Acid Substitution: The replacement amino acid is not chosen uniformly at random. Instead, the algorithm queries the MSA or LLR matrix to select a substitute residue probabilistically (line 12). This ensures that the new residue is evolutionarily plausible according to the data-driven models.
6. Structural Refinement: The mutation is applied using the pyRosetta *mutate_residue()* function (line 13). Subsequently, a *local_fast_relax()* protocol is executed to relieve any steric clashes and accommodate the new side chain within the structural environment, ensuring the variant is physically realistic.

**Algorithm 1** Mutation Operator Algorithm**Require:** $Pop_{g},\;L,\;mut\_rate,\;MSA\_Matrix,\;LLR\_Matrix$  1:**for** $indiv\in Pop_{g}$ **do**  2:       aa_st, aa_unst←get_list_of_st_unst_aa(indiv, L)    ▹ interface_energy  3:       **if** size(aa_unst)≠0 **then**  4:             size_aa←size(aa_unst)  5:       **else**  6:             size_aa←size(aa_st)  7:       **end if**  8:       max_mutations←size_aa×mut_rate  9:       num_mutations←random(1,max_mutations)10:       **while** n_mut<=num_mutations **do**11:             aa, position←SelectAAPosition(aa_st∨aa_unst)12:             aa_r←ChooseReplacementAA(aa, MSA_Matrix∨LLR_Matrix)13:             Mutate_Residue(indiv, aa_r, position)        ▹ pyRosetta function14:             local_fast_relax(indiv)      ▹ Constrained Rosetta FastRelax mover15:             n_mut←n_mut+116:       **end while**17:**end for**

#### 2.5.4. Objective Functions:

We define two complementary objective functions to guide the search:

**f1: energy_score (Physics-based)**: This study utilizes the $\Delta G_{separated}$ metric, measured in Rosetta Energy Units (REU), as the primary criterion for thermodynamic stability. It is computed using the Rosetta *InterfaceAnalyzer* application [16], which characterizes protein–protein interfaces by calculating binding energies and buried surface areas. The optimization goal is to minimize this objective, defined as:

(1) $$energy\_score=\frac{dG_{separated}}{dSASA}\cdot 100$$

where $dG_{separated}$ represents the calculated binding free energy (the difference in energy between the complexed state and the separated chains), and dSASA is the change in Solvent Accessible Surface Area (SASA) upon binding (buried interface area in ${Å}^{2}$). Consequently, Equation (1) represents the binding energy density (energy per unit of interface area), providing a normalized measure of interaction efficiency.

**f2: ΔLog Likelihood Ratios—LLR (Data-driven)**: Mutations in protein sequences can range from deleterious to neutral or beneficial. Assessing these functional effects is challenging; however, protein language models like ESM-2 offer powerful proxies for fitness by learning evolutionary constraints. We utilize the Log-Likelihood Ratio (LLR) as a metric for sequence plausibility. The change in likelihood is expressed by Equation (2):

(2) $$\Delta LLR=\sum_{i\in M}logP\left(x_{i}=x_{i}^{mt}|x_{-M}\right)-logP\left(x_{i}=x_{i}^{wt}|x_{-M}\right)$$

where *M* represents the set of mutated positions, *P* is the conditional probability predicted by the model for amino acid $x_{i}$ at position *i*, $x_{i}^{mt}$ is the mutant-type residue, and $x_{i}^{wt}$ is the wild-type residue. A positive value (ΔLLR>0) indicates that the variant sequence fits the learned evolutionary model better than the wild type, suggesting a potentially stabilizing or more natural mutation. The optimization goal is to maximize this objective.

### 2.6. Computational Evaluation Metrics

To rigorously assess the thermodynamic quality of the redesigned interfaces beyond the optimization objectives, we evaluated several physicochemical metrics using the Rosetta *InterfaceAnalyzer* application. These indices provide a comprehensive view of the binding feasibility and structural complementarity of the solutions:

- Energy Score ($f_{1}$): The primary physics-based objective function (defined in Equation (1)). It represents the binding energy density, normalizing the interaction strength by the interface size.
- $\Delta G_{binding}$ (dG_separated): An approximation of the binding free energy in Rosetta Energy Units (REU). It is calculated as the energy difference between the complexed state and the separated chains. Lower (more negative) values indicate stronger affinity.
- ΔSASA (dSASA_int): The change in Solvent Accessible Surface Area upon binding, measured in ${Å}^{2}$. This metric quantifies the buried interface area, serving as a proxy for the extent of molecular contact.
- Packstat: A dimensionless packing statistic (0.0–1.0) that evaluates the volumetric complementarity of the interface residues. Values above 0.65 typically indicate a well-packed, crystal-quality interface [16].
- Interface Energy: The sum of residue-pair interaction energies across the interface, providing a direct measure of enthalpic stability.

### 2.7. Experimental Setup

To evaluate the proposed framework, we designed three distinct experimental scenarios focused on the redesign of the Rs551 bacteriophage RBP complex:

1. **Experiment 1:** Single-Objective Optimization (Minimizing $f_{1}$: energy_score) utilizing the MSA Matrix to guide the mutation operator.
2. **Experiment 2:** Single-Objective Optimization (Minimizing $f_{1}$: energy_score) utilizing the LLR Matrix (ESM-2) to guide the mutation operator.
3. **Experiment 3:** Multi-Objective Optimization (Minimizing $f_{1}$ and Maximizing $f_{2}$: ΔLLR) utilizing the LLR Matrix for mutation guidance.

Experiments 1 and 2 are designed to isolate and compare the impact of different data-driven guidance methods (MSA vs. ESM-2) on thermodynamic stability. Experiment 3 represents the full hybrid approach, seeking to fuse physical stability with biological plausibility. The population size, number of generations, and mutation rate were determined empirically. A mutation rate of 0.3 achieved the best balance between exploration and exploitation: lower values caused premature convergence and reduced diversity, while higher values disrupted good solutions and slowed convergence. The evolutionary parameters were maintained consistent across all experiments to ensure comparability:

- Search Space (*N*): 38 interface residue positions (defined by Face C).
- Population Size: 25 individuals.
- Generations: 20.
- Mutation Rate: 0.3.
- Independent Runs: 16 per experiment.

## 3. Results and Discussion

### 3.1. Algorithm Convergence

**Figure 3:**
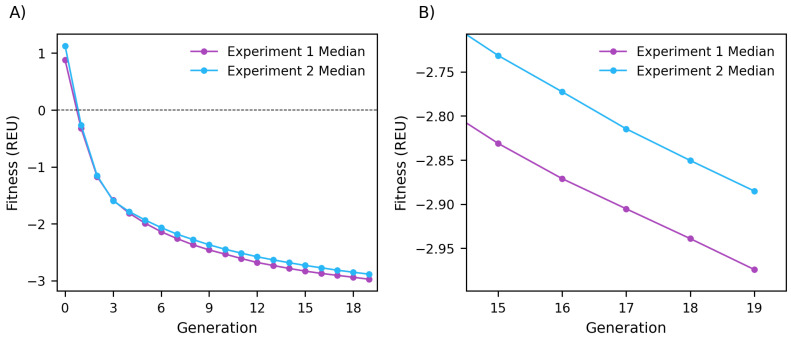
(**A**) Algorithm Convergence for Experiments 1 and 2. (**B**) Convergence in last five generations.

We assess the optimization trajectory by monitoring the average fitness metric across all independent runs. Figure 3 depicts the progression of average fitness over the course of the generations. The horizontal axis represents the generation count, while the vertical axis displays the average binding energy in Rosetta Energy Units (REU). The dashed line indicates the zero-energy threshold; values below this line represent favorable thermodynamic interactions. Figure 3 illustrates that as the generations progress; the median fitness value decreases significantly. Notably, both Experiments 1 and 2 (Figure 3A) exhibit rapid convergence, achieving stability within just 20 generations.

Our primary objective in these initial experiments was to evaluate the efficacy of the mutation operator when guided by local evolutionary history (MSA, Experiment 1) versus deep learning-derived patterns (ESM-2, Experiment 2). While both approaches yield substantial improvements in the energy score, the MSA-guided operator exhibits a slight performance advantage (Figure 3B). This difference is likely attributed to the fact that MSA explicitly leverages evolutionary constraints from homologous sequences that are structurally identical to the target RBP. In contrast, ESM-2 draws from a generalized protein language model trained on the entire protein universe, providing broad but less target-specific guidance for this particular complex.

To validate the multi-objective formulation, we analyzed the correlation between ΔLLR and energy_score for all solutions generated in Experiment 2. The analysis yielded a negligible correlation coefficient of −0.003. This confirms that thermodynamic stability (energy) and sequence plausibility (LLR) are distinct, non-redundant criteria. Consequently, in Experiment 3, we treated binding energy (minimization) and ΔLLR (maximization) as independent objectives. The mutation operator, guided by ESM-2, explores the sequence space by identifying variants that simultaneously optimize physical stability and evolutionary likelihood.

**Figure 4:**
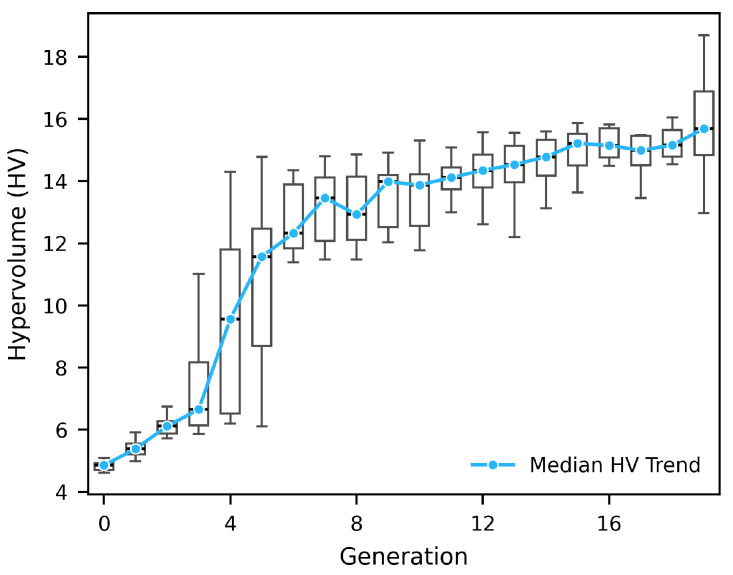
Algorithm Convergence for Experiment 3 (Hypervolume Evolution). Boxplots represent the hypervolume distributions across all 16 runs per generation. The blue line connects the median hypervolume values.

We assessed the convergence of the multi-objective approach (Experiment 3) using the hypervolume metric [29], which quantifies the volume of objective space dominated by a solution set relative to a reference point. Figure 4 presents the hypervolume distributions across all 16 runs. The consistent upward trend in the median hypervolume indicates a progressive improvement in the quality and diversity of the Pareto approximation throughout the optimization process. The variability observed in the boxplots suggests that while all runs improve, the exploration of the trade-off surface varies stochastically between independent executions.

**Figure 5:**
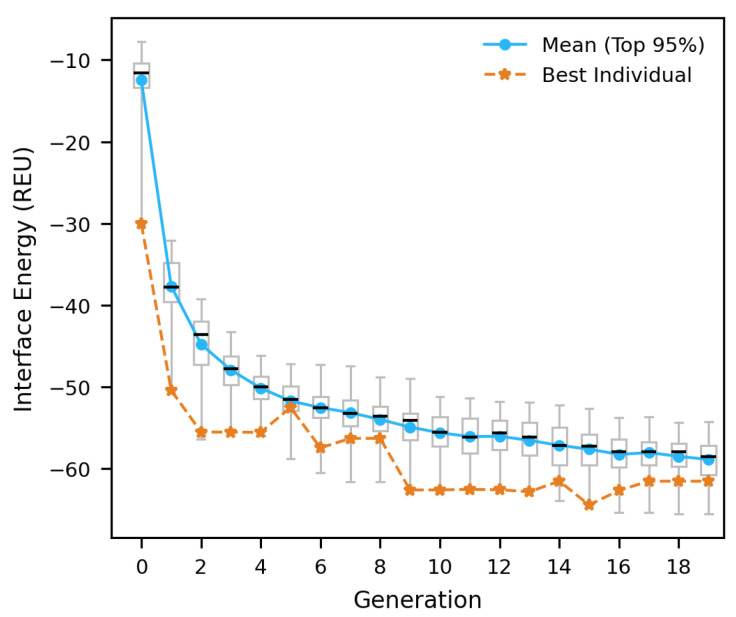
Energetic Optimization Trajectory. The boxplots illustrate the dispersion of the Top 100 individuals per generation (Mean trend in blue). The orange dashed line tracks the interface energy of the best individual discovered in each generation.

To provide a granular view of the energetic optimization trajectory, we tracked the distribution of the top 100 solutions and the single best individual across generations using the interface_energy program that calculates the pairwise energetic contribution of each amino acid at the interface. Figure 5 illustrates this convergence profile. The analysis reveals a robust optimization behavior characterized by a steep descent in the initial generations, followed by asymptotic refinement.

Statistically, the system demonstrates high efficiency. The mean interface energy of the top-performing population improved drastically from −12.44 REU in Generation 0 to −58.87 REU in Generation 19, representing a relative improvement of over 370%. The spread of the boxplots (interquartile range) significantly narrows after Generation 5, indicating that the population successfully converged toward a basin of energetic stability.

Crucially, the “Best Individual” trajectory (orange dashed line) leads the population, starting at −30.04 REU and reaching a global minimum of −64.44 REU at Generation 15. The proximity between the population mean (−58.87) and the best individual (−61.53) in the final generation confirms the algorithm’s ability to not only find a single optimal solution but to propagate favorable stabilizing mutations across the entire population. The linear regression analysis yields a negative slope of −1.31 REU/generation for the population mean, quantitatively validating the consistent downward trend in binding energy.

### 3.2. Solutions

**Figure 6:**
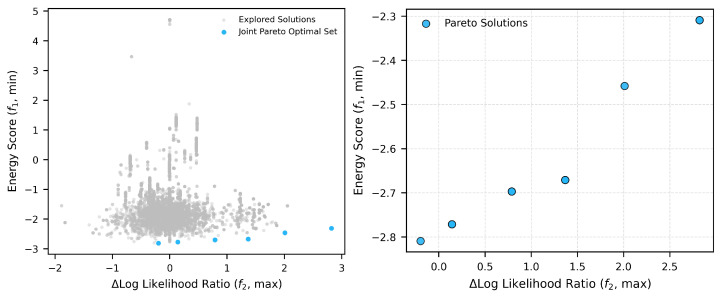
Pareto Optimal Set (POS). (**Left**): Optimization Landscape showing all explored solutions. (**Right**): Detailed Pareto Optimal Frontier.

For the analysis in this section, we aggregate all solutions from the 16 independent runs and compute the non-dominated set from this combined pool. This unified set, termed the Pareto Optimal Set (POS), represents the best trade-off solutions discovered by the algorithm for Experiment 3. Figure 6 displays the Pareto front corresponding to the POS solutions as blue dots. On the left side, the plot displays the POS along with all solutions, on the right side, only the solutions belonging to the POS are shown.

To further analyze the diversity and conservation of amino acid choices among the optimal solutions, we constructed a frequency matrix using all sequences from the POS. In this matrix, each cell represents the frequency with which a specific amino acid appears at a given sequence position across the POS. Rows correspond to amino acids, while columns indicate sequence positions, allowing us to visualize patterns of variability and conservation within the set of optimal solutions.

**Figure 7:**
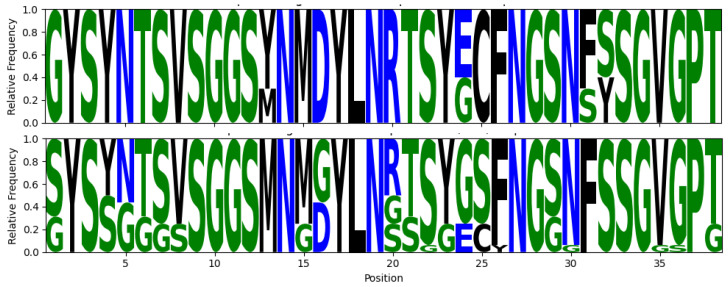
Sequence Logo at G0 (**Top**) and POS (**Bottom**): Colors residues based on hydrophobic vs. hydrophilic character. Hydrophobic (A, V, I, L, M, F, W, Y) in dark tones, Polar in greenish, Charged in blue. The top sequences come from the initial population at generation zero (G0). The bottom sequences come from the Pareto Optimal Set (POS).

The matrix can be visualized by graphical representations using sequence logos [30,31]. Logos are composed of characters arranged in layers, each represented by integer values. The height of each character signifies its biological importance [32]. The downside of Figure 7 displays the sequence logo generated from the frequency matrix data obtained from POS. The horizontal axis represents the 38 index positions used in our analysis, while the vertical height of each character indicates its relative frequency derived from the frequency matrix.

The downside of Figure 7 shows that positions 2 and 3 are exclusively occupied by the amino acids Y and S, respectively, as indicated by their relative frequencies of 1, meaning that every solution in the POS contains these specific residues at those locations. Additionally, there are regions, such as positions 9 to 14 and 31 to 34 in which no variation occurs across all optimal solutions, thereby, emphasizing highly conserved segments. In contrast, several other positions display considerable diversity, with multiple amino acids represented in different layers according to their observed frequencies among the solutions. In this way, we can observe the mutation rates that have occurred in the POS for all positions.

We can verify which zones have changed relative to the initial population. Figure 7 compares the solution obtained from the initial population (Generation Zero, top) with those obtained in the Pareto Optimal Set (POS, bottom).

Note that there are some invariant positions where the amino acids remain the same during evolution. Stable and unstable amino acids guide the mutation operator, resulting in invariant position regions showing high stability. In contrast, certain position segments exhibit a high number of mutations. For instance, positions 4–9 have seen an increase in both the number and proportion of amino acids compared to the initial generation. Another region displaying similar behavior is located between positions 15–16, 20–23, 29–30, 35–36. Additionally, an interesting behavior regarding initial generation is noted. In some positions, the mutation effect and the evolution process replace a set of amino acids with a single one, such as in position 13 or 31. In position 24, the proportion of amino acids is inverted compared to the initial generation.

### 3.3. Binding Affinity and Interface Analysis

To rigorously assess the thermodynamic quality of the redesigned interfaces, we evaluated several biophysical metrics using the Rosetta *InterfaceAnalyzer* and *interface_energy* application. We conducted a two-tiered analysis: first, examining the global thermodynamic descriptors of the complex, and second, deconstructing the interaction energy at the residue level to trace specific evolutionary trajectories.

#### 3.3.1. Global Thermodynamic Metrics

Table 1 summarizes the statistical comparison between the initial random population (G0) and the evolved POS. The data reveal an important improvement in thermodynamic stability across all energy-related indices.

The optimization efficacy is evidenced by the shift in the mean Interface Energy, which transitioned from a repulsive state in G0 (+32.14 REU) to a highly attractive state in the POS (−60.54 REU). A critical insight is provided by the standard deviation of $\Delta G_{binding}$, which collapsed from 434.31 in G0 to just 4.66 in the POS. This indicates that the evolutionary process successfully filtered out deleterious variants with severe steric clashes, converging the population into a narrow, stable energy funnel. Regarding structural packing, the Packstat score remained stable (∼0.70), and ΔSASA showed only a slight reduction (∼8%). This behavior is consistent with the *local_fast_relax* protocol employed, which restricts backbone flexibility to the vicinity of mutations. The algorithm optimized affinity by maximizing specific side-chain interactions within the available volume rather than by inducing large-scale conformational changes.

#### 3.3.2. Residue-Level Evolutionary Trajectories

**Figure 8:**
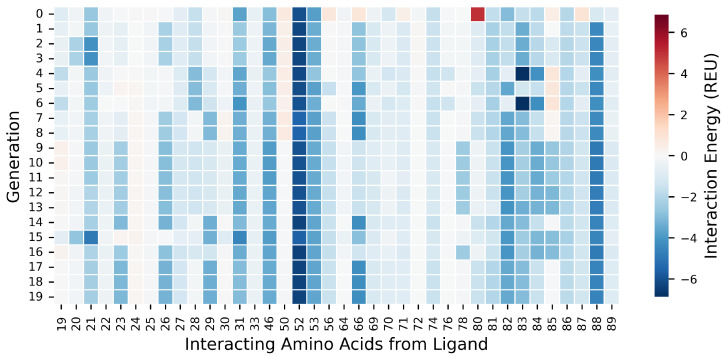
Per-Residue Interface Energy Decomposition. The heatmap tracks the interaction energy (REU) of each ligand residue (x-axis) across 20 generations (y-axis). Blue indicates favorable attraction, while red indicates repulsion. Columns such as 52 and 88 represent stability anchors, while columns 66 and 80 show successful energetic rescue events.

To understand the specific molecular drivers of this affinity maturation, we analyzed the per-residue energy contribution of the best-performing individuals, according to the energy_score function, across generations (Figure 8). This granular analysis reveals that the complex’s thermodynamic stability relies on a core set of stability anchors, which are residues that serve as the structural foundation of the interface. Residue 52 acts as the primary anchor, maintaining a deep favorable energy (Mean: −6.07 REU) with minimal variance (σ=0.27) throughout the entire process. Similarly, positions such as 53, 88, and 46 exhibit consistently high affinity (Mean < −3.3 REU) and low fluctuation. The evolutionary search effectively identified and conserved these energetic hotspots early in the trajectory, protecting them from deleterious mutations that could destabilize the complex.

Beyond preservation, the algorithm demonstrated a remarkable capacity for adaptive optimization. The most important improvements occurred at positions initially plagued by steric clashes or electrostatic repulsion. For instance, Residue 80 underwent a critical transformation, shifting from a highly repulsive state (+5.03 REU) to a stabilizing contribution (−1.51 REU), yielding a net energetic gain of over 6.5 REU. A similar trajectory is observed for Residue 66, which evolved from a liability (+0.80 REU) into a top contributor (−4.27 REU). Furthermore, residues with high plasticity, such as 23 and 29, exhibited the steepest evolutionary slopes (Slope < −0.15), indicating continuous fine-tuning to maximize complementarity. These dynamic adjustments drive the global energy descent shown in Figure 5, confirming that the algorithm operates by simultaneously locking in structural pillars while aggressively remodeling suboptimal regions.

## 4. Conclusions

In this study, we presented a hybrid computational framework fusing physics-based and data-driven methods to optimally redesign bacteriophage proteins for enhanced interaction with bacterial receptors. Our methodology leveraged elitist single-objective and multi-objective evolutionary algorithms to navigate the complex energetic landscape of the protein interface. The decision variable space encompassed the interfacial region, comprising thirty-eight distinct residue positions. To explore this space, we implemented a specialized mutation operator that targets energetically suboptimal residues and stochastically selects replacements guided by homologous sequence data (MSA) and deep learning-derived probabilities (ESM-2).

We first evaluated the mutation operator under a single-objective formulation to minimize interaction energy. The comparison between MSA-guided and ESM-guided strategies revealed that both effectively direct the search toward stable regions, with minimal performance differences. Subsequently, we deployed a multi-objective evolutionary algorithm to simultaneously minimize binding energy and maximize the log-likelihood ratio. This approach proved highly effective, as the integration of physics-based stability and data-driven plausibility facilitated the discovery of the Pareto Optimal Set. The results demonstrate that specific, targeted amino acid substitutions can drastically reduce the protein–protein interaction energy (improving affinity) while maintaining evolutionary coherence. Crucially, our analysis elucidated the functional impact of these mutations, identifying invariant residues essential for stability and variable positions that modulate binding affinity.

In future work, we aim to incorporate greater structural flexibility by relaxing the local backbone constraints and exploring novel adaptive mutation strategies. We also plan to expand the multi-objective framework by integrating compromise programming indices to automatically select the most balanced designs from the Pareto front. To further validate the computational designs and ensure their biological relevance, subsequent studies will include experimental validation through in vitro and in vivo assays, focusing on the binding affinity, stability, and functionality of the redesigned protein–protein complexes. This experimental phase will bridge the gap between computational predictions and real-world applications, confirming the practical viability of the optimized designs derived from our evolutionary algorithms and hybrid modeling approach. While this work focuses on the biological control of pathogenic bacteria in agriculture, the proposed protein redesign strategy is transferable to other biomedical applications. Ultimately, we intend to validate these computational findings through experimental assays, synthesizing the redesigned RBP variants to engineer bacteriophages with superior bactericidal efficiency and expanded host ranges.

## Figures and Tables

**Table 1:** Comparison of Interface Metrics: Initial Population (G0) vs. Pareto Optimal Set (POS). Format: “Mean (G0)|Mean (POS)”.

Metric	Min (G0|POS)	Max (G0|POS)	Mean (G0|POS)	StdDev (G0|POS)
energy_score ($f_{1}$)	−0.57|−2.81	4.70|−2.30	1.06|−2.60	0.86|0.19
$\Delta G_{binding}$	−13.01|−61.75	113.35|−49.64	25.32|−56.96	434.31|4.66
ΔSASA	2268.79|2150.39	2452.99|2241.97	2381.11|2188.76	25.55|31.33
Packstat	0.62|0.63	0.79|0.77	0.71|0.69	0.03|0.05
Interface Energy	−30.03|−63.20	75.13|−54.33	32.14|−60.54	17.41|3.80

## Data Availability

The code and data are available in the next git repository: https://github.com/applibres/protein_ea_02/tree/feat/localrelax (accessed on 31 January 2026).
